# High-stakes examinations during the COVID-19 pandemic: to proceed or not to proceed, that is the question

**DOI:** 10.1136/postgradmedj-2020-139241

**Published:** 2021-01-15

**Authors:** Chee-Kiat Tan, Wei-Ling Chua, Charles Kien-Fong Vu, Jason Pik-Eu Chang

**Affiliations:** Gastroenterology and Hepatology, Singapore General Hospital, Singapore; Joint Committee on Specialist Training, Academy of Medicine Singapore, Singapore; Gastroenterology and Hepatology, Tan Tock Seng Hospital, Singapore; Gastroenterology and Hepatology, Singapore General Hospital, Singapore

**Keywords:** medical education & training, gastroenterology

## Abstract

The COVID-19 pandemic has disrupted education-related activities, including the conduct of examinations. We review the literature regarding high-stakes examinations during the pandemic, discuss the decision-making process of whether to proceed with a high-stakes examination and share published experiences in conducting high-stakes examinations during the pandemic. We illustrate our own recent experiences of decision-making and conduct of our high-stakes gastroenterology licencing examinations during the height of the COVID-19 pandemic. It is possible to conduct high-stakes examinations virtually during pandemic situations with fidelity and adherence to the established format and standards.

## Introduction

The COVID-19 pandemic came suddenly and swiftly. Stringent measures are imposed to decrease the spread of the disease. Safe distancing is one of the mainstays in our fight against COVID-19.^1^

Not unexpectedly, medical education, including examinations, is disrupted. The teaching faculty is also busy with clinical duties during this emergency situation. Social distancing makes it very challenging to conduct clinical examinations. Hence, during the current COVID-19 pandemic, there are many instances of cancellation of high-stakes national and graduating professional examinations.^2–4^ Final-year medical students in Italy are fast-tracked into the medical workforce without sitting for the hitherto mandatory practical examinations.^3^ The British Medical Association (BMA) supports the delay of non-essential examinations to avoid risk to students and teachers and diversion of hospital resources.^5^ Similarly, some medical schools in the UK do not conduct high-stakes final examinations and instead allow their final-year medical students to proceed on to work in the hospitals to help their national health system cope with the COVID-19 crisis.^6^ However, a high-stakes final-year examination is to guarantee society that the medical school delivers competent doctors.^7^ Hence, there will be concerns about the impact on patient care when final-year examinations are bypassed. Nevertheless, a new way of life in medical education has become inevitable.^8 9^ Though we are now cognisant of preventive measures against COVID-19 transmission and vaccines are on the horizon, possibly allowing the resumption of high-stakes examinations in 2021, we are currently in the third wave and lockdowns and there is no telling when the pandemic will end.

Similarly, there are concerns about conducting high-stakes qualifying exit examinations in postgraduate medical education during the COVID-19 pandemic. Even though the pandemic situation in Singapore is not as dire as in the UK and Italy, the peak of our community spread occurred during the period of high-stakes medical examinations. In this article, as members of the gastroenterology licencing examination board, we look at the issue of high-stakes examinations during the COVID-19 pandemic, integrating our experience with our recent high-stakes gastroenterology licencing examination.

## Decision-making about examinations during the COVID-19 pandemic

Any examination conducted during the pandemic must maintain the usual standards. The BMA acknowledges that this is a challenging task.^5^ There must be careful consideration about reasons to persist with the scheduled examination and whether the existing robust examination methodology can still be applied during this pandemic period.^10^ A practical way to approach this is to apply the ‘what, why, when, how’ framework.^11^ This framework addresses the purpose and necessity of the examination as well as the timing and format of the examination. Throughout the decision-making process, adherence to standards and validity of the examination are critically important.

### What

What is the examination about? What is it for? Is it an important or non-essential examination? Is the examination for maintenance, progression, exit or licencing? In our case, the examination is a high-stakes licencing examination for our specialty trainees who have successfully completed their 3-year Accreditation Council for Graduate Medical Education International accredited gastroenterology training. In Singapore, all graduating gastroenterology specialty trainees need to pass a mandatory national licencing examination sanctioned by the Ministry of Health before they can practise as independent gastroenterology specialists.

What will the format of the examination be? It should ideally be the same as previous prepandemic years for the best assurance of validity and maintenance of standards. If possible, there should not be any new methods of testing that are unfamiliar to the candidates and examiners as the time available for them to adapt to these new methods may be unrealistic.^12 13^ The pandemic itself already significantly impacts the confidence and preparedness of candidates taking high-stakes examinations without any changes in the examination formats.^14^ A change in the examination format is even more unsettling. Hannon *et al* described how they converted their end of clerkship examination from the usual in-person objective structured clinical examination (OSCE) to remote OSCE.^15^ More than half of the candidates felt that the remote OSCE could not assess clinical skills as well as in-person OSCE and they also felt awkward about the way the remote examination was conducted. Indeed, there is a need to develop online faculty development modules for the examiners on the new methods of testing.^16^ The format of our gastroenterology licencing examination in Singapore consists of a MCQ paper and a viva voce. As neither component involves patients, we were able to retain the same format during the pandemic. This instilled confidence in the maintenance of standards and validity. We acknowledge that it is less challenging to organise an examination under social distancing rules when there are no patient contact components. However, others have shown that it is also possible to organise a high-stakes examination that includes in-person OSCE with patients.^17 18^

### Why

Why do we need to conduct the examination now when there is currently a severe strain on the healthcare system, on doctors and on hospitals? Our gastroenterology specialty board feels that we cannot abolish the examinations as it is an important licencing examination for qualification as full-fledged specialists in our medical system. Society expects us to impose the highest standards for our specialists. Most importantly, we feel that we can conduct the examination with the same rigour and format as previous years despite the current medical emergency in the country and with minimal additional strain on our healthcare system. One could also consider the drawbacks of not having the high-stakes exit examination. The abolishment of high-stakes qualifying examinations in Italy is feared to have repercussions on patient care^3^

### When

Does the examination need to be conducted now? If it is a replacement assessment, is there sufficient time for the candidates and examiners to familiarise themselves with any new processes/systems? Is it possible to conduct a high-fidelity clinical examination with mandatory social distancing? Intuitively, one would think that with mandatory social distancing and universal precautions against infection, the conduct of clinical examinations is a big challenge. The reasons why we did not defer our licencing examination include (1) the negative impact on our national supply of new gastroenterologists, (2) the delay in the career progression of our specialty trainees and (3) the disruption of new specialty trainees coming into the gastroenterology training programme. In addition, our examination format does not require patient contact.

### How

How is the examination going to be conducted? Will there be new processes with novel problems? Will disparities in WIFI access or electronic devices cause uneven fairness of the examination? With the need for social distancing, the default mode of testing would be remote. High-stakes examinations do not have remote assessment as the norm. As remote assessment involves technology and new processes, we must be mindful that these can potentially disadvantage less technology-savvy candidates. When introducing technology into an examination, there can be three possible outcomes (1) no difference in the intellectual process, (2) there is a difference but does not change the score achieved and (3) the difference changes the score achieved.^19^ To ensure validity and fidelity of the examination, the technology used should not cause a significant difference in the intellectual process or in the score achieved. In addition, to be equitable and fair, there should be an equivalent technological environment for everyone, including candidates with disabilities.^20^ One must be careful not to be technology-centric but instead should remain candidate-centric.^13^ There is an excellent discourse by Hillier and Fluck on high-stakes e-examinations (ie, online examinations) examining various issues, requirements, problems and solutions for conducting high-stakes examinations remotely.^21^ It is key to avoid pedantry over software and hardware and instead focus on establishing and adhering to best practices. Examples of best practices include provision of all candidates with an equivalent environment, availing students of a dummy examination for practice and provision of user guides.

## Our experiences in conducting a high-stakes licencing examination in gastroenterology in Singapore during the COVID-19 pandemic

We have described how, based on the ‘what, why, when, how’ framework, we decided to proceed with our high-stakes licencing examination in gastroenterology during the COVID-19 pandemic. Our gastroenterology licensing examination comprises MCQs and a viva voce. The MCQs were done with traditional pen and paper as in previous years. The candidates were socially distanced by being secluded in separate individual rooms during the duration of the MCQ test. The viva voce examination was conducted in four different rooms within the examination venue itself and used a video conferencing software on equivalent laptop set ups. Thus, there was an equivalent technological environment for all the candidates. An information technology (IT) technician was on standby within the premises throughout the duration of the examination. Experience from others has shown that technology hitches are common.^15^ All the examiners were in their own private offices in their respective hospitals. Thus, the candidate and the two examiners were all isolated from each other. The candidates and examiners were all very familiar and adept with video conferencing software as they have been using it for online meetings and teaching sessions since the pandemic started more than 3 months ago. As in previous years, each candidate was examined on two viva voce questions with two different examiners per question, marking on a common scoring rubric. This format is identical to that in all previous years except that it is now conducted remotely via video conferencing software.

The factor that is very important to our considerations and final decision is that as a national licencing examination, it must be robust and maintain the same standard and validity as in the previous years. That we are able to retain the same examination format is an important assurance. As described by Hartley *et al* and Mogey *et al*, we are also confident that the inevitable introduction of technology into this year’s examination will not result in a change of intellectual processes or cause undue unfairness and disadvantage to any of the candidates.^19 20^ It is known that modifications to a high-stakes examination held during the COVID-19 pandemic may reduce the validity of the assessment method.^18^ The fact that the MCQ was not a remote test but still the same traditional pen and paper test as in previous years assured us about the validity although it can be argued that nowadays candidates are becoming increasingly accustomed to MCQs conducted and validated in an electronic format. As the viva voce was held in the same venue with the same technology setup for all the candidates, all of whom were able-bodied, there was no undue fairness or disadvantage to any particular candidate. Other best practices that we adopted included comprehensive briefing sessions for the candidates, live rehearsals a week before the examination to test the connectivity between the examination venue and the examiners in their own hospital offices and ensuring coexaminers for the viva voce are from different hospitals to reduce the likelihood of both examiners having connectivity issues at the same time. As the viva voce was conducted in a similar format to previous years with the same examiners and scoring scheme, the validity and standards of the viva voce examination were maintained. The only difference was that this was now conducted remotely using video conferencing software. However, as all the candidates and examiners were very adept in using video conferencing software, we were confident that the candidates’ intellectual processing and the examiners’ assessment and marking abilities would not be affected. All these factors assure us that the standards and validity of this high-stakes qualifying examination will be maintained.

As expected, organising and conducting this year’s examination during the COVID-19 pandemic was more challenging. We required 88 more man-hours than previous years. The additional man-hours were due to the coordination with more agencies, testing the video conferencing software at the examination venue with the examiners in their own offices, additional invigilators as the candidates were in their own individual rooms for the MCQ paper and an onsite IT technician on the day of the examination. On the day itself, there was a technical hitch resulting in a loss of communications for 2 min in two of the rooms for viva voce. Two candidates were affected and they were compensated with another 2 min for the viva voce. Since they did not fail the viva voce and the examination was a criterion-referenced and not a norm-referenced examination, we felt that the interruption did not unfairly affect the outcomes. Nevertheless, the loss of connection could have caused emotional distress as there would be a loss of train-in-thought that may not have been easily compensated with the two additional minutes. We are cognisant that technical hitches may have impact on candidates.^15^

**Figure 1:**
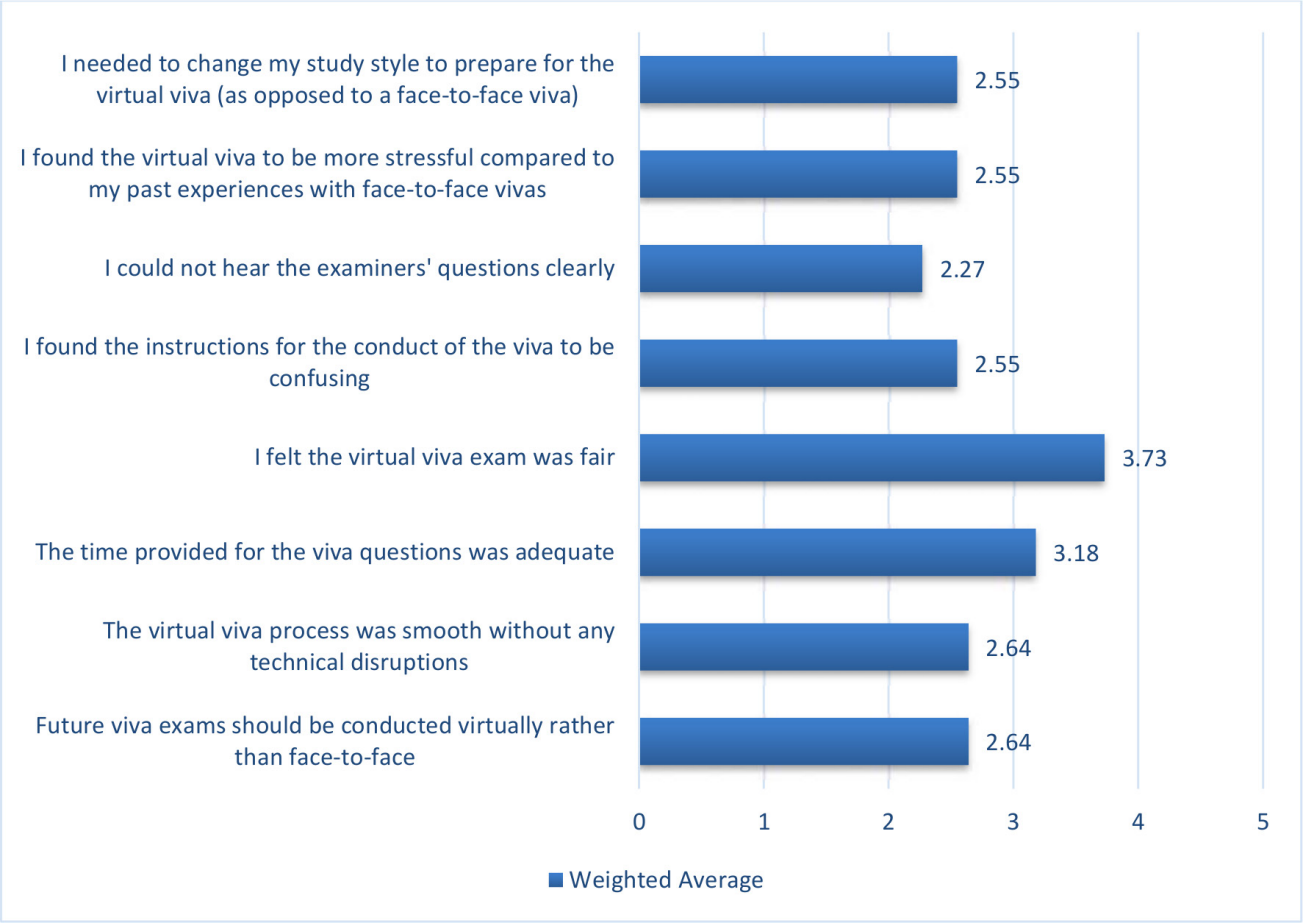
Survey results of candidates.

**Figure 2:**
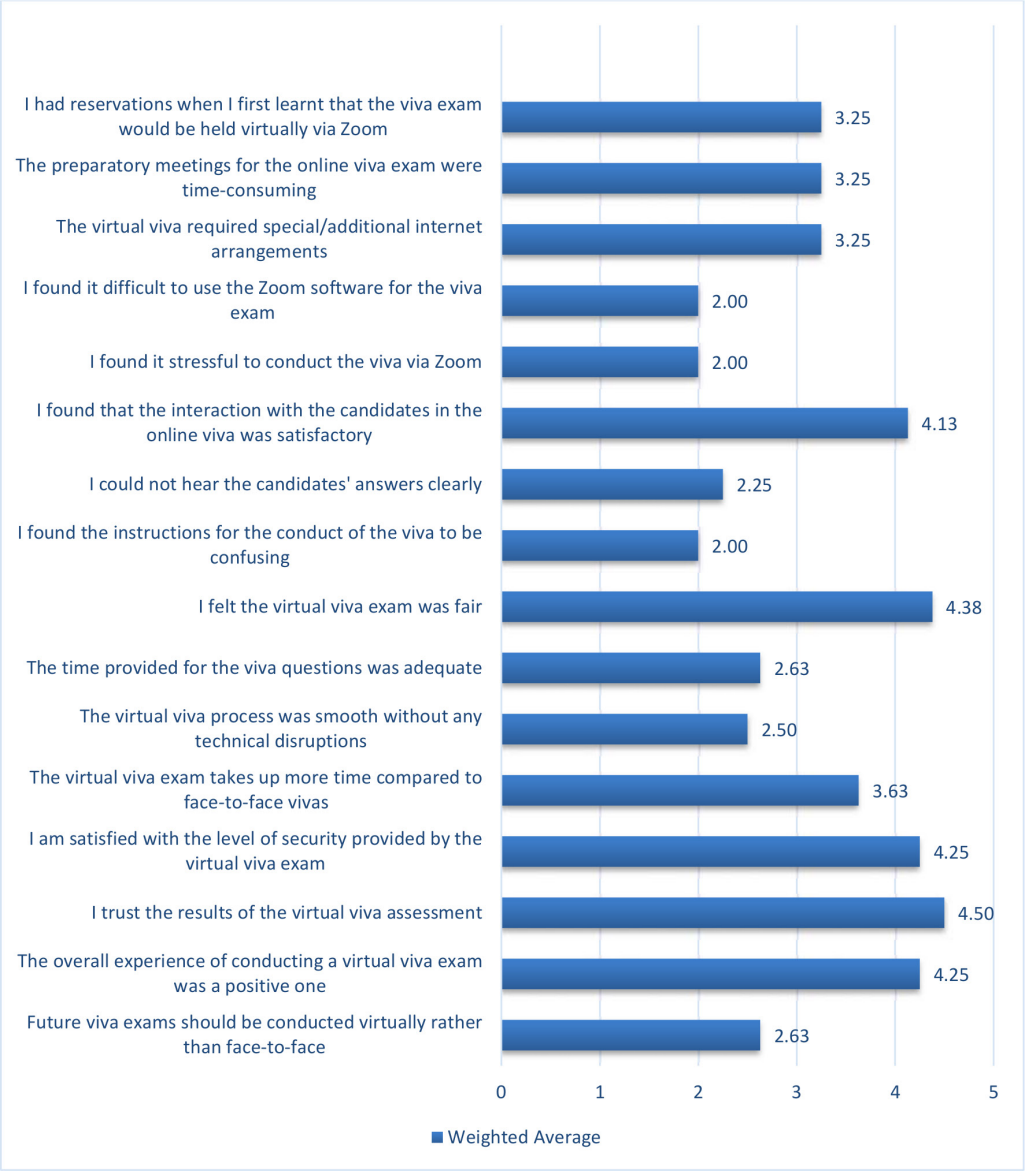
Survey results of examiners.

We also wanted to know the candidates’ and examiners’ experiences and sentiments about the high-stakes examination being held with a novel remotely conducted viva voce during a pandemic. Hence, we conducted an anonymised web-based survey with all the 11 candidates and eight examiners after the examination. The survey questions were all based on a 5-point Likert scale ranging from strongly disagree (1)to strongly agree (5). The questions encompassed perceptions before the examination and sentiments about the actual examination. The results showed that both candidates and examiners were satisfied with the conduct of the examinations (figures 1 and 2). Importantly, the candidates found that the examimation to be fair and the examiners trusted the reliability of the examination outcome. Stoopler *et al* had also reported positive experiences with their remotely conducted structured viva voce non-high-stakes examination for one of their dental school modules.^22^ All our eight examiners have been examining for the past few years. Thus, they are in a position to attest that this year’s examinations remained the same format and that they have examined with the same rigour as in previous years. These contributed to their opinion that this year’s examination was fair and that they trust the results of the examinations (figure 2). This is an important indication of the maintenance of standards and the robustness of our high-stakes gastroenterology licencing examination despite being conducted semiremotely during the COVID-19 epidemic.

## Reported experiences of conducting high-stakes examinations during the pandemic

To our knowledge, there is no publication in the literature on the conduct of a high-stakes gastroenterology examination. There is a publication on a high-stakes orthopaedic clinical examination during the COVID-19 pandemic.^23^ Malhotra *et al* reported on the positive experience with their orthopaedic residency exit examination held during the COVID-19 pandemic. As patient contact was prohibited, they prepared several sets of clinical case studies, pictures and radiographs that were shown digitally to the candidates. The exit examination was not held remotely but had candidates donning N95 facemasks and gloves and physically distanced from the examiners. The overall satisfaction was high for both examiners and candidates.

Boursicot *et al* recently reported a successful high-stakes OSCE for their final-year medical students during the COVID-19 pandemic.^17^ Their reason for holding the high-stakes examination during the pandemic was to ensure their final-year medical students graduate on time to enter and bolster the healthcare workforce. All the patients were confined to their own separate room throughout the day-long examination. Students were physically distanced at all times. Examiners’ briefing and calibration were via video conferencing software on individual iPads. All these describe their iteration of the ‘what, why, when and how’ framework. Pandemic-related issues included having to recruit new clinician examiners on short notice because the regular examiners were deployed to frontline patient care, having to use video conferencing because clinicians from different hospitals were not allowed to physically interact and apprehension over whether their external examiners from UK and Australia were still able to travel.

Samarasekera *et al* shared briefly about their experience with a high-stakes clinical examination for their final-year medical students. They used a clinical case scenario instead of patients, observed all infection control precautions and safe distancing and minimised the number of examiners.^18^ Importantly, they made special efforts to reassure the students that there will be a special makeup examination for those who are unwell, quarantined or infected so that students who were unwell were not tempted to turn up for the high-stakes examination.

Some medical schools held their examinations remotely with the candidates in their own homes.^24^ However, this is not ideal as equity of remote examinations depends on all students having a similarly suitable environment at home to take the examination including efficiency of internet access and impact of technical problems.^25^ We chose to have all our candidates be physically present at the examination venue to ensure examination security as well as an equivalent technological environment for all the candidates.

## Conclusion

A framework to guide decision-making is important when considering whether to proceed with a high-stakes examination during the COVID-19 pandemic. There are reports of successful conduct of high-stakes clinical examinations during the pandemic while fully observing all personal protection measures and safe distancing. We have shown that despite daunting challenges, the current state of technology is able to successfully support a high-stakes examination conducted remotely. The most important point is that despite special measures and processes due to the COVID-19 pandemic, we are still able to retain the format and maintain the validity and standard of the examination. These are especially important in high-stakes examinations. In addition, our candidates and examiners are able to embrace new technology. This augurs well as technology is playing an increasingly important role in medical education.

**List of learning points**

- A decision-making framework is important when considering whether to conduct a high-stakes examination during a pandemic.
- The use of technology is inevitable in a pandemic. Besides ensuring an equivalent technological environment for all candidates, it is also important to ensure that this introduction of technology does not impede the usual intellectual processes of the candidates or affect the performance of a candidate.
- We need to be sure that the assessment is fair, accurate and conform to previous standards of the high-stakes examination.
